# Uncovering Proteomic and Biochemical Alterations in Plasma from Lesch–Nyhan Disease Patients

**DOI:** 10.1007/s10571-025-01644-z

**Published:** 2025-12-15

**Authors:** Sundas Javed, Daniela Braconi, Haidara Nadwa, Alessandro Paffetti, Gabriella Jacomelli, Vanna Micheli, Barbara Marzocchi, Annalisa Santucci, Giulia Bernardini

**Affiliations:** 1 Department of Biotechnology, Chemistry and Pharmacy, University of Siena, Siena, Italy; 2 LND Famiglie Italiane ODV, Via Giovanetti 15-20, Genova, Italy; 3 UOC Laboratorio Patologia Clinica, Ospedale S. Maria alle Scotte, AOU Senese, Siena, Italy

**Keywords:** Plasma proteomics, Cytokines, Systemic inflammation, 2D-PAGE, Hyperuricemia, Purine salvage pathway

## Abstract

Lesch–Nyhan disease (LND) is an ultra-rare X-linked inborn error of metabolism caused by complete or partial deficiency of hypoxanthine-guanine phosphoribosyltransferase (HPRT), a key enzyme in the purine salvage pathway. This defect leads to uric acid overproduction and a broad spectrum of neurological and behavioral manifestations, whose severity depends on the degree of residual enzymatic activity. Although emerging evidence implicates HPRT deficiency in widespread cellular dysfunctions, particularly within midbrain dopaminergic neurons, the molecular mechanisms underlying the neurobehavioral phenotype of HPRT deficiency remain poorly understood and are not adequately explained by purine metabolism dysfunctions alone. Although proteomics represents a powerful approach for elucidating molecular alterations underlying disease, it has so far found only limited application in LND research. To address this gap, we provide here the first proteomic study combined with clinical biochemistry data and pro-inflammatory cytokines profiling of plasma samples from 29 HPRT deficient individuals (21 with classic LND and 8 with Lesch–Nyhan variants – LNV). We suggest that plasma proteomics might be a potential tool in LND for monitoring disease progression and therapeutic response, potentially paving the way for targeted treatment strategies that extend beyond the purine salvage pathway to address the currently unmet clinical needs of LND patients.

**Graphical Abstract:**

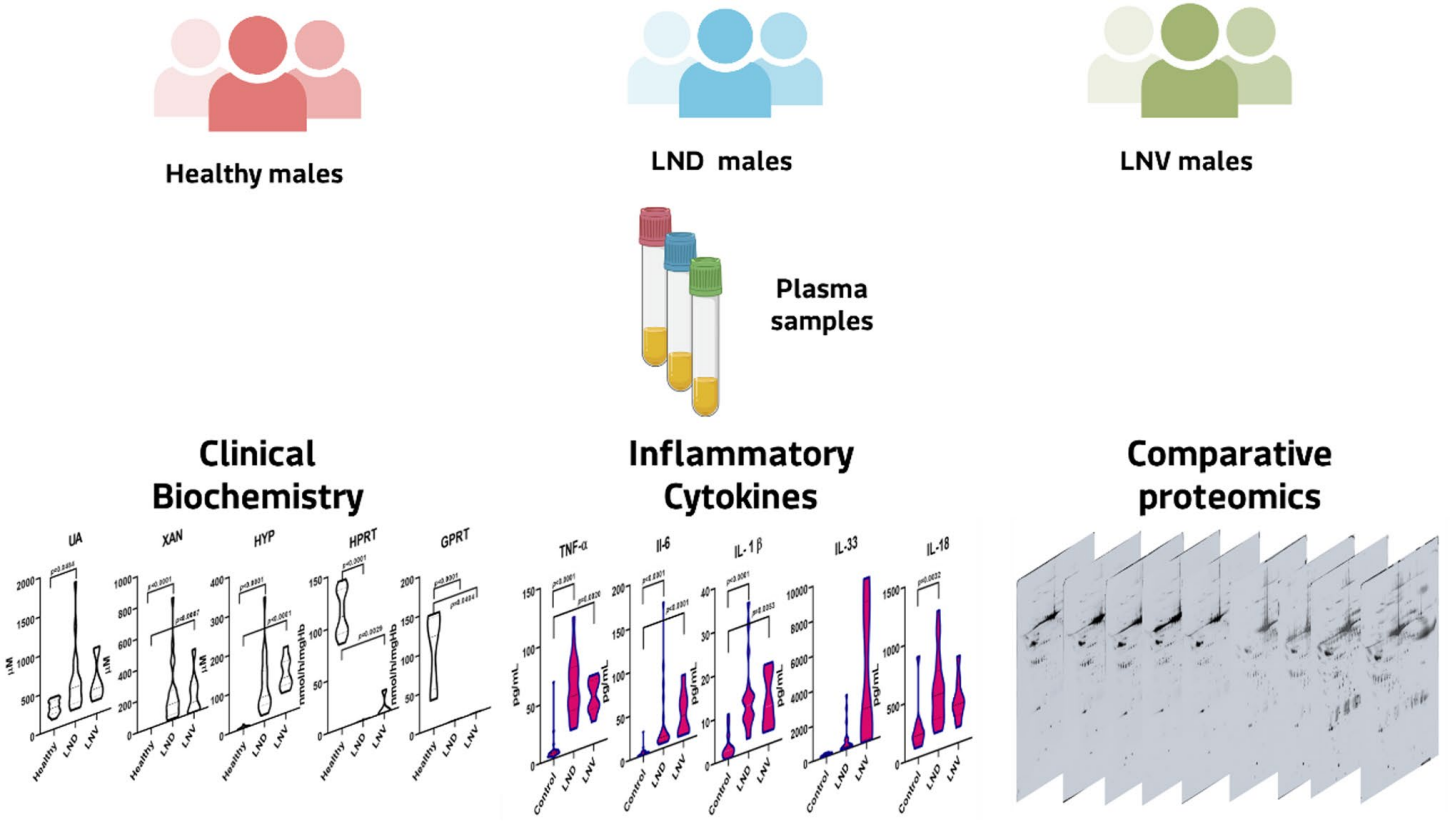

**Supplementary Information:**

The online version contains supplementary material available at 10.1007/s10571-025-01644-z.

## Introduction

Lesch–Nyhan disease (LND; OMIM 308000) is a X-linked recessive disorder associated with a defective hypoxanthine-guanine phosphoribosyltransferase (HPRT; EC 2.4.2.8) activity caused by pathogenic variants in the *HPRT1* gene. The disorder is rare, with a variable reported incidence across different populations and geographic regions ranging from 1 in 235,000 to 1 in 500,000 live births globally (Torres 2019). HPRT is a critical enzyme in the purine salvage pathway (Micheli et al. 2020; Camici et al. 2023; Javed et al. 2024) whose deficient activity (complete or partial) leads to the accumulation of purine metabolites, mainly uric acid (UA) and hypoxanthine (HYP), in blood, urine, and cerebrospinal fluid (Torres and Puig 2007).

HPRT deficiency causes a multisystemic disorder characterized by hyperuricemia and hyperuricosuria, associated with a complex array of neurological and behavioral patterns, including dystonia, choreoathetosis, cognitive impairment, and a peculiar self-injurious behavior (SIB) that evolves over time and depends on the severity of the enzymatic defect (Fu et al. 2014; Micheli et al. 2020; Camici et al. 2023). Affected individuals are typically normal at birth but begin to exhibit clinical symptoms within the first years of life. Based on residual HPRT enzyme activity and the presence or absence of specific neurological features, three major clinical phenotypes have been defined (Fu et al. 2015). The classical LND phenotype (residual enzyme activity 0–1.5%) is associated with severe motor and cognitive impairment (100% and 95% of prevalence, respectively) and SIB (100% of prevalence). Partial phenotypes, or Lesch–Nyhan variants (LNV), encompass two milder forms: HPRT-related neurologic dysfunction (HND, residual enzyme activity 1.5–8.5%), characterized by motor impairment and neurocognitive abnormalities (91% and 67% of prevalence, respectively) and without SIB; and HPRT-related hyperuricemia (HRH, residual activity > 8%) in which neurological symptoms are absent or minimal (Christie et al. 1982; Jinnah et al. 2010).

Diagnosis relies on both clinical and laboratory data. In newborns, hyperuricemia and hyperuricosuria, either isolated or with psyco-motor delay, warrant enzymatic testing in erythrocytes or fibroblasts, followed by genetic testing (Torres and Puig 2007).

Despite significant advances in elucidating the genetic and biochemical basis of LND, the precise molecular mechanisms underlying its neurological and neurobehavioral manifestations remain poorly understood (Javed et al. 2024) and are not adequately explained by purine metabolism dysfunction alone. Multiple lines of evidence suggest that dysfunction of dopaminergic signaling, mitochondrial impairment, and disrupted protein homeostasis contribute to the pathogenesis of HPRT deficiency. In vivo clinical studies have shown that the neurological manifestations are linked to dysfunction of basal ganglia dopamine systems (Jinnah et al. 2006), whereas ex vivo analyses of post-mortem human brain tissue and in vivo studies in HPRT-deficient mouse models have revealed reduced tyrosine hydroxylase expression without neuronal loss, indicating an aberrant dopaminergic phenotype (Göttle et al. 2014). More recently, in vitro studies using patient-derived neural stem cells have demonstrated that HPRT deficiency disrupts mitochondrial function and protein homeostasis, further implicating these pathways in abnormal neural development (Dinasarapu et al. 2022). Dysfunction of midbrain dopaminergic (DA) neurons is believed to be a key contributor to the neurobehavioral manifestations of LND, involving abnormalities in both DA neuron development and function. In vivo studies in HPRT-deficient mouse models have demonstrated altered gene expression in dopaminergic regions of the brain (Song and Friedmann 2007) and developmental abnormalities in midbrain DA neuron proliferation, migration, and organization (Witteveen et al. 2022). Complementary in vitro and ex vivo investigations using HPRT-deficient neuronal and neural stem cell models have shown dysregulation of transcription factors critical for dopaminergic differentiation (Ceballos-Picot et al. 2009; Cristini et al. 2010; Guibinga et al. 2010; Sutcliffe et al. 2021). and deficits in neurite outgrowth, supporting a neurodevelopmental origin of DA dysfunction. Furthermore, since neurological symptoms are refractory to current UA-lowering treatments, a deeper understanding of LND pathophysiology (Torres and Puig 2015; Torres 2019) and associated biochemical and proteomic alterations could help the identification of novel potential therapeutic targets extending beyond the purine salvage pathway.

In this context, proteomic approaches offer a promising avenue to uncover disease-related molecular signatures. Proteomics, enabling the identification of significant changes in protein abundance, post-translational modifications, and protein-protein interaction networks, can provide systems-level insights into disease biology (Aebersold and Mann 2016). In rare diseases, where limited sample availability is a significant challenge, proteomics can thus maximize data yield from minimal sample volumes (Braconi et al. 2021, 2025). In the case of LND, the proteomic characterization of patient-derived samples, such as plasma or serum, could provide valuable information about systemic changes in protein expression and signalling pathways associated with the disease. Plasma, in particular, represents an accessible and informative matrix for proteomics-based biomarker discovery owing to its capacity to reflect both local and systemic physiological alterations (Braconi et al. 2021, 2025). However, current research into LND has largely focused on UA and purine-related metabolites (Ceballos-Picot et al. 2015; Madeo et al. 2019) whereas there has been very limited interest into the proteomic characterization of LND patients’ biofluids.

To address this gap, in this study we present a comprehensive biochemical and proteomic analysis of plasma samples from 29 HPRT deficient individuals (21 with classic LND and 8 with LNV). By integrating proteomic profiling with conventional biochemical analyses, we aim to provide novel insights into the systemic molecular changes associated with LND, identifying dysregulated proteins and biological pathways that may underlie the complex neurobehavioral manifestations of LND. Furthermore, we aim to explore the potential of plasma proteomics as a tool for monitoring disease progression and therapeutic response. Our findings may advance the understanding of LND and pave the way for the development of targeted treatment strategies to address the currently unmet clinical needs of patients with this devastating disorder.

## Materials and Methods

### Subjects and Samples

All LND subjects participating in this study were diagnosed according to currently accepted criteria (Jinnah et al. 2006, 2010). Because HPRT deficiency is inherited as an X-linked recessive disorder, all cases were males. Blood from LND male subjects was drawn by venipuncture and sent to our laboratory for diagnostic purposes and, after informed consent and in accordance with the declaration of Helsinki, agreed to donate their blood samples for this study. The study received approval from the Local Ethics Committee. Samples were collected in fasting conditions in clean tubes containing heparin as anticoagulant. After centrifugation, blood cells were used fresh for the evaluation of enzyme activities, and plasma samples were separated and kept frozen at − 80 °C until analysis. According to biochemical diagnosis (residual HPRT enzymatic activity) and clinical symptoms observed at the time of sampling, subjects were dived into two groups: 21 classic LND and 8 LNV. Plasma samples from age-, ethnicity-, and sex-matched male subjects with normal HPRT enzyme activity were selected and used as controls for cytokines evaluation. For 2D-PAGE analysis, a commercially available pooled healthy human male plasma was used as a control. Demographics and clinically relevant information on patients selected for the study are schematically reported in Table 1.

### Clinical Biochemistry

Purine metabolites (UA, HYP and xanthine (XAN) in plasma extracts were measured by HPLC-UV as described previously (Jacomelli et al. 2019). To produce acidic extracts, 0.6 N perchloric acid was added to plasma (1:1) and mix thoroughly. The samples were centrifuged at maximum speed for 5 min and the supernatants were transferred into clean tubes and were brought to neutrality by 3.5 M potassium carbonate. Samples were centrifuged at maximum speed for 5 min just before HPLC analysis.

Biochemical characterization also included HPRT and GPRT activity in erythrocyte lysates, when available (Jacomelli et al. 2019). Red cells were washed twice with isotonic NaCl (155 mM). Cell-free lysates were obtained from washed erythrocytes by water dilution (1:5) and freezing–thawing and then centrifuged at maximum speed for 10 min and the supernatants were transferred into clean tubes. Assay mixtures for H/GPRT contained 55 mM Tris buffer pH 7.4, 5.5 mM MgCl_2_, 1 mM phosphoribosyl-1-pyrophosphate, 0.3 mM αβ-methylen-ADP, 0.6 mM either HYP, or guanine and 200 µl of cell homogenate in a final volume of 200 µl; sample were incubated at 37 °C for 20 min. Reactions were stopped by one volume of 0.6 N perchloric acid; after centrifugation (12,000 × g for 2 min) clear supernatants were brought to neutrality by 3.5 M potassium carbonate. Samples were centrifuged at maximum speed for 5 min just before HPLC analysis. Hemoglobin (Hb) quantification was used for data normalization.

The HPLC apparatus consisted of a Beckman System Gold Module125S, with amod.168 Nouveau diode array detector. Phenomenex Luna C18 columns (3 μm particle size, 75 × 4.6 mm) equipped with guard columns (Phenomenex Security guard 4 × 3 mm) were used. The HPLC analyses were performed by gradient elution using 10 mM potassium phosphate, pH 5.5, and methanol as eluant, at room temperature, with 1 ml/min flow rate, and absorbance monitoring at 260 and 280 nm. Peak identities were confirmed by retention time, coelution with added internal standards, 260/280 nm absorbance ratios, and UV/vis spectra. Quantification was based on concentration/peak area linear plots developed injecting solutions with known concentrations of each pure compound.

### Cytokines Quantification

Assessment of plasma concentrations of IL-1β, IL-6, IL-18, IL-33 and TNF-α was performed via commercially available multiplex bead-based immunoassay kits (EMD Millipore Corporation) as per the manufacturer’s protocols. The detection range was 0.49–2,000 pg/ml for IL-1β, 0.18–750 pg/ml for IL-6, 0.43–1750 for TNF-α, 19.5–20000.5 for IL-33 and 4.9–5000 for IL-18. Quantification of analytes was obtained using spline curve-fitting methods generated with appropriate standards (Braconi et al. 2022).

### 2D-PAGE and Image Analysis

50 µg of proteins/sample were submitted to 2D-PAGE and silver ammoniacal staining as described previously (Lorenzetti et al. 2015; Braconi et al. 2016). Digitalized images were obtained with ImageS-canner III and analyzed by ImageMaster software (GE HealthcareBioSciences). The increasing/decreasing index (fold-change) was calculated as the ratio of spot% relative volume between gel maps; for multiple spots identified as different molecular species of a same protein, the total % relative volume was used for calculations. Protein spot identification was carried out by gel matching with the master gel of human plasma retrieved by http://world-2dpage.expasy.org/swiss-2dpage.

## Statistical and Bioinformatics Analysis

The experiments were carried out in triplicate. Results were processed through GraphPad 8.0. Normal distribution was analyzed with D’Agostino-Pearson or Shapiro Wilk test depending on sample size, and descriptive statistics were obtained for each analyzed dataset. Non-parametric variance (Kruskal–Wallis) of the median and non-parametric *post hoc* (Dunn’s) tests were used due to the non-normal distribution of any variable in any group. For 2D-PAGE data, statistically significant thresholds of 2.0 and 0.5 for fold-change values in protein relative abundance ratios calculated as LND/control were set to highlight over-represented and under-represented proteins, respectively. Gene Ontology (GO) enrichment analysis was performed using STRING web service (https://string-db.org/) with the differentially expressed proteins revealed by proteomic analysis used as input. Significantly enriched pathways were retrieved by searching against KEGG and REACTOME databases (Szklarczyk et al. 2023).

## Results and Discussion

### Clinical Biochemistry

We assessed several pathologically relevant biochemical markers in the plasma of 21 LND, 8 LNV, and 20 healthy, age-matched controls (Tables 1 and 1S). About 71% (15 out of 21) of the LND subjects and 38% (3 out of 8) of the LNV subjects were under UA-lowering therapy with allopurinol. The tested markers included: levels of UA, HYP and XAN, and the activity of HPRT and GPRT enzymes. As expected, statistically significant higher plasma levels of HYP and XAN were found in LND and LNV compared to controls (Table 1).

**Table 1:** Clinical biochemistry of LND (21) and LNV (8) individuals and healthy controls (20)

Marker	Healthy (*N* = 20)	LND (*N* = 21)*	LNV (*N* = 8)*	*p* value^#^
*AU (µM)*				
Median	275.8	437.6	322.7	**0.0484** ^a^ 0.6425^b^ ˃0.9999^c^
25–75%	200.8–363.7	234.5–620.1	223–716.8	
*HYP (µM)*				
Median	2.36	61.68	91.65	**˂0.0001** ^a^ **˂0.0001** ^b^ ˃0.9999^c^
25–75%	1.08–4.33	40.99–127.80	71.91–132.10	
*XAN (µM)*				
Median	0.525	104.7	65.85	**˂0.0001** ^a^ **0.0007** ^b^ ˃0.9999^c^
25–75%	0.38–0.95	35.97–190.5	6.163–178.7	
*HPRT (nmol/h/mgHb)*				
Median	107.1	0	0.03	**˂0.0001** ^a^ **0.0029** ^b^ 0.6232^c^
25–75%	91.25–133.7	0–0	0.002–16.50	
*GPRT (nmol/h/mgHb)*				
Median	117.5	0	0.07	**˂0.0001** ^a^ **0.0404** ^b^ 0.7372^c^
25–75%	50.03–142.3	0.00–0.00	0–0.29	

Descriptive statistics (median and interquartile range) are reported for plasma metabolites (UA, HYP, XAN) and enzyme activities (HPRT, GPRT) in healthy age-matched controls, LND, and LNV subjects. Comparisons among groups were performed using the Kruskal–Wallis test followed by Dunn’s correction for multiple comparisons

^#^*p-values*: ^a^healthy vs. LND, ^b^healthy vs. LNV, ^c^LND vs. LNV. Statistically significant p-values (<0.05) are reported in bold. *About 71% (15 out of 21) of the LND subjects and 38% (3 out of 8) of the LNV subjects were under UA-lowering therapy with allopurinol

### Inflammation Markers

Growing evidence suggests that inflammatory cytokines play a pivotal role in neurogenesis, and high levels of pro-inflammatory cytokines have been associated with alterations in cognitive and learning processes in various neuropsychiatric disorders, such as depression, and neurodegenerative diseases, including Alzheimer’s disease and Parkinson’s disease (Borsini et al. 2015).

**Fig. 1:**
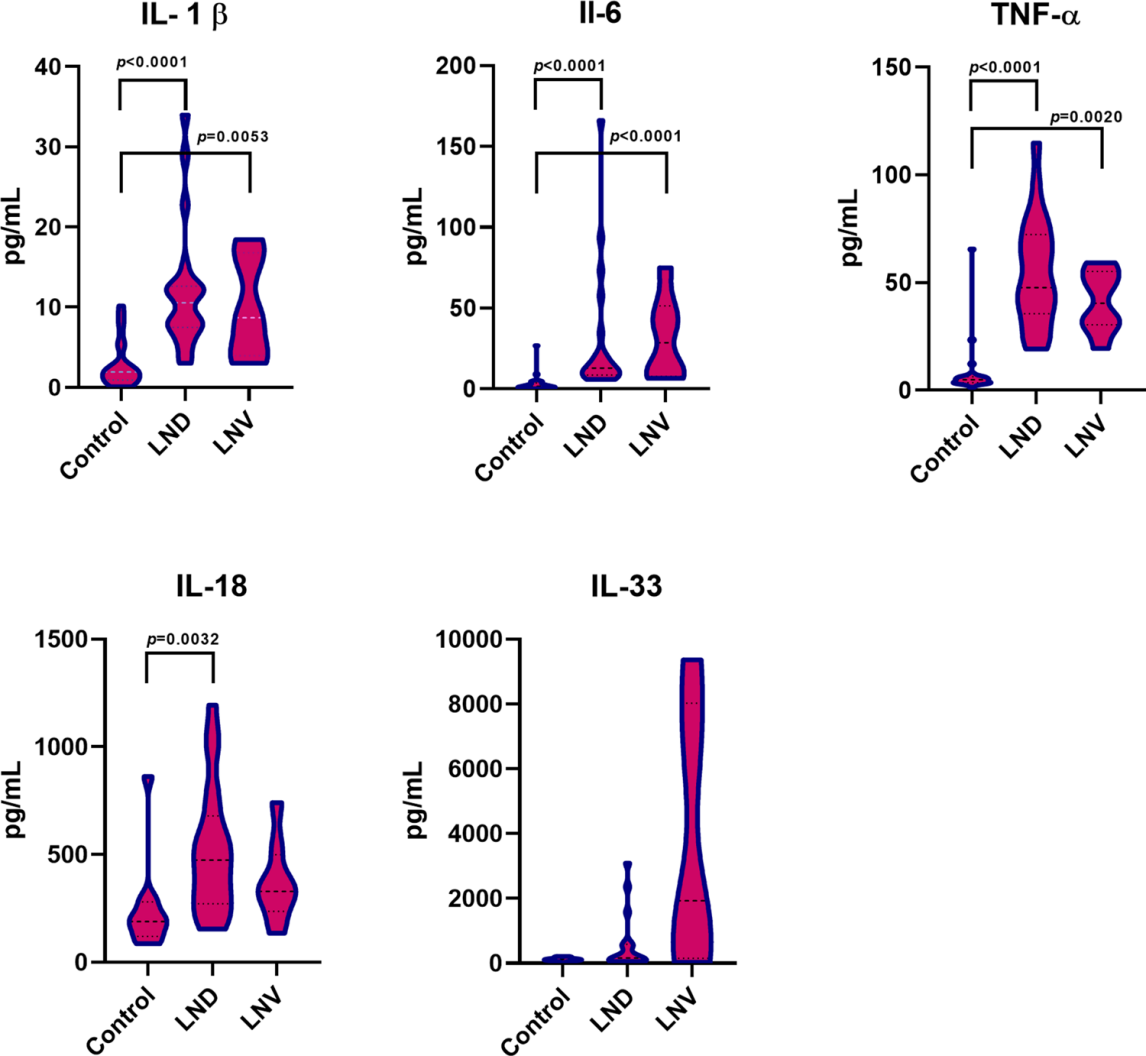
Cytokine levels (IL-1β, TNF-α, IL-6, IL-18, and IL-33) in healthy controls, LND, and LNV subjects. Violin plots illustrate the distribution and median values of cytokine concentrations across groups. Statistical comparisons were performed using the Kruskal–Wallis test, followed by Dunn’s post hoc test for multiple comparisons. Significant differences between groups are indicated where applicable. *p-values* < 0.05 were considered as statistically significant

To investigate the potential involvement of inflammation in LND, we assessed plasma levels of five pro-inflammatory cytokines - IL-1β, TNF- α, IL-6, IL-18, and IL-33 - in our samples. We found statistically significant higher levels of IL-1β, IL-6, TNF-α and IL 18 (but not IL-33) in LND and LNV samples compared to healthy controls (Fig. 1 and Table 2S).

Notably, none of the analyzed inflammatory markers showed statistically significant differences between patients with the classic LND phenotype (characterized by profound neurological impairment including SIB) and those with LNV phenotype (typically associated with milder or even absent neurological symptoms and no SIB). This suggests that the current binary classification into LND and LNV may be too broad to capture subtle, yet potentially meaningful, biological differences among patients. A more granular phenotypic stratification, possibly integrating biochemical indicators, including residual HPRT enzyme activity and baseline UA levels (prior to hypouricemic therapy), together with quantitative neurological assessments based on standardized clinical scales, may provide greater discriminatory power.

We then performed correlation analyses to assess whether the levels of inflammatory biomarkers were influenced by patients’ age or biochemical parameters. We found statistically significant inverse correlations between patients’ age and both IL-18 (ρ=−0.700, *p* < 0.0001) and TNF-α (ρ=−0.560, *p* = 0.002).

**Fig. 2:**
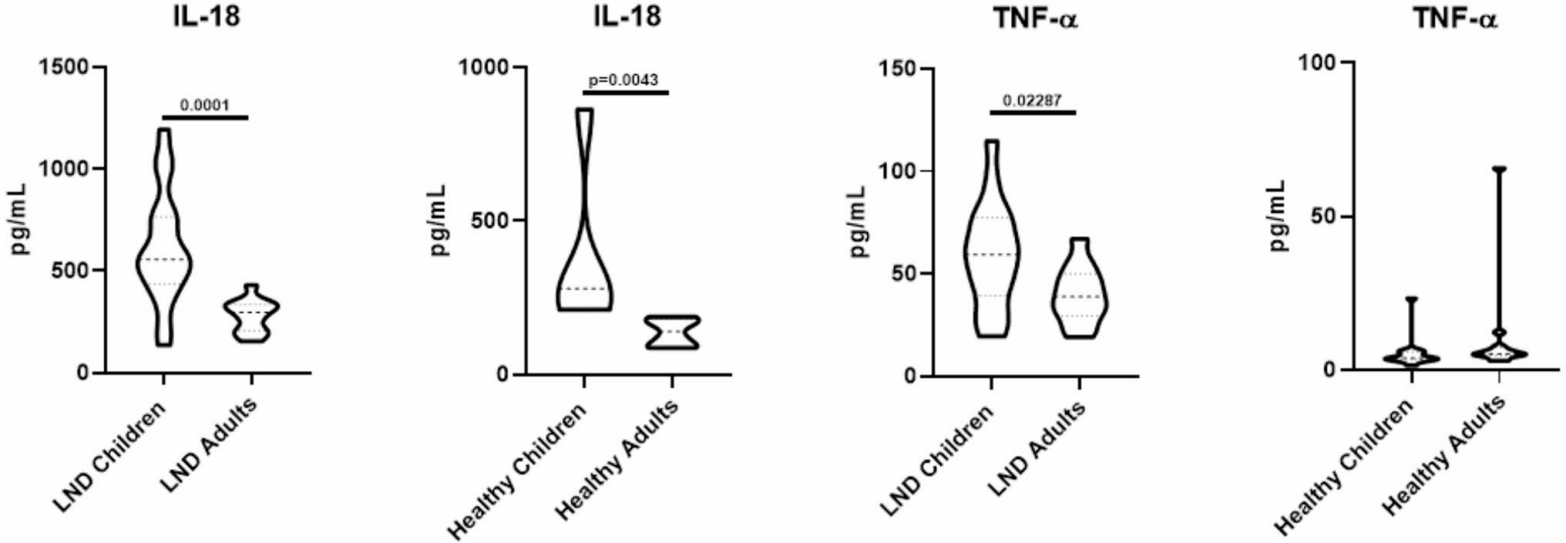
Age-related distribution of plasma cytokines TNF-α and IL-18 in LND. Violin plots show the distribution and median levels (pg/mL) of TNF-α and IL-18 in LND children (≤ 14 years) and LND adults (> 14 years). Statistical significance was determined with Two-tailed Mann-Whitney test

To further investigate the role of patients’ age on these two inflammatory markers, we performed a direct comparison of TNF- α and IL-18 levels between healthy and LND subjects grouped by age (children ≤ 14 years and LND adults > 14 years), as reported in Fig. 2. The decrease of IL-18 levels in both LND and healthy adults indicates that the observed decline may reflect a normal physiological age-related trend rather than a disease-specific effect, consistent with previous findings (Kleiner et al. 2013). In contrast, TNF-α levels appeared to decrease with age only within our LND patient cohort, potentially reflecting progressive disease-related alterations in inflammatory regulation over time.

The potential impact of long-term UA–lowering therapy on inflammatory cytokine levels was also assessed; however, no statistically significant differences were observed for any of the analysed markers (Fig. 1S).

Furthermore, our data suggest that an inflammatory milieu may appear very early in LND. Nearly 60% of our plasma samples were from individuals younger than 14 years, and elevated cytokine levels persisted across all the stages of psychological, cognitive, and motor development.

Cytokines are integral to the structural and functional maturation of the central nervous system. Emerging evidence shows that pro‑ and anti‑inflammatory mediators modulate the neurogenic niche by influencing the proliferation and differentiation of neuronal progenitor cells, thereby shaping neurodevelopmental trajectories (Stolp 2013; Borsini et al. 2015). In the context of LND, early and sustained cytokine dysregulation could therefore have profound implications for brain development and contribute to the neurobehavioral phenotype observed in these patients.

### Comparative Proteomics

**Fig. 3:**
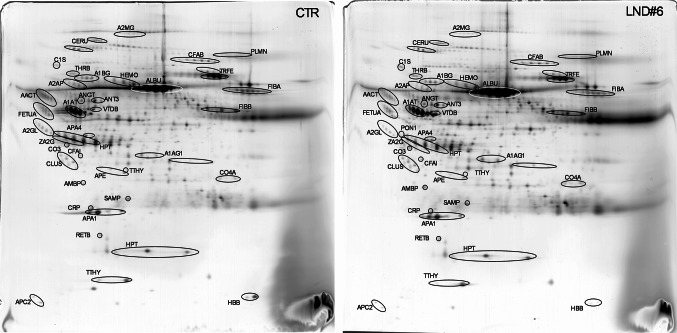
Comparative proteomics. Silver stained 2D maps of a representative LND plasma sample (right) and healthy control plasma (left). The quali-quantitative analysis carried out allowed the identification of differently expressed proteins, which are indicated with their abbreviated names and described in Table 2. Relative abundance of protein spots is reported in Fig. 3. Only representative images from a triplicate set are shown

**Fig. 4:**
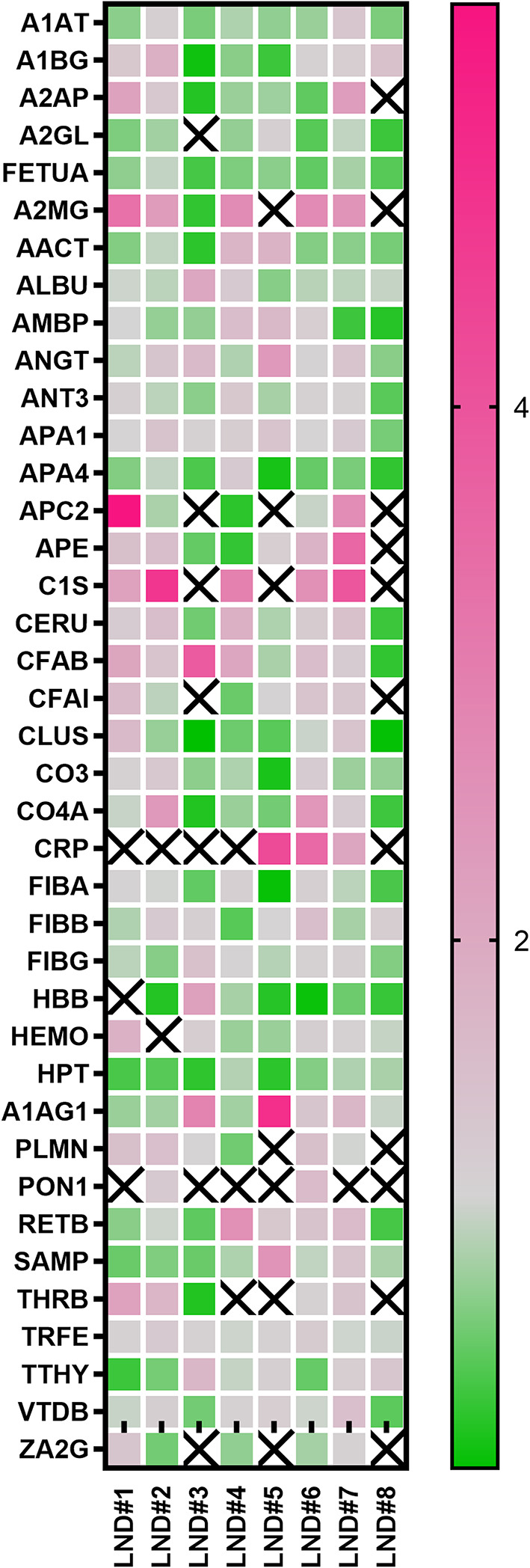
Heat map of differentially expressed proteins in LND plasma versus control. Each column represents one of the eight LND samples, and each row corresponds to a protein whose abundance was found to be significantly altered compared to controls. Color intensity reflects the fold-change values, with green indicating down-regulation, pink indicating up-regulation, with the gradient indicating the magnitude of change

A small subset of eight LND samples (characterized by elevated levels of UA, HYP and XAN and null HPRT activity) was thus selected for a comparative proteomics analysis against a commercially available pool of healthy plasma. Samples were also matched in terms of age, ethnicity, and sex (males). All LND samples were under treatment with allopurinol. We employed a classical two‑dimensional gel electrophoresis workflow without depletion of abundant proteins to preserve the native proteome composition, thereby generating an “unbiased snapshot” of each sample. An average of 613 ± 92 spots were resolved in each proteomic map, and about 220 spots were identified per map, corresponding to 57 different proteins. Quantitative comparative analysis between LND and control samples revealed similar expressions patterns for a set of proteins, including: apolipoprotein A-I (APOA1), apolipoprotein C-III (APOC3), apolipoprotein D (APOD), the alpha subunit of the hemoglobin (HBA), histidine-rich glycoprotein (HGR), several species and subunits of immunoglobulins (IGHA, IGHG, OGHM, IGLC, IGSA, IGSG, IGSM), the gamma chain of fibrinogen (FIBG), serotransferrin (TRFE) and alpha-1-antitrypsin (A1AT). Conversely, all the remaining identified proteins showed a statistically significant differential expression in LND sample(s) relative to controls. These differentially expressed proteins are annotated in Fig. 3 and schematically reported with their main features in Table 2. A detailed presentation relative abundance changes is provided in Fig. 4; Table 2.

**Table 2:** Proteins differently expressed in LND samples respecting to healthy controls

AN^a^	Protein	Entry	Functions	spot% rel vol		fold-change LND/CTR							
				range CTR	range LND	LND#1	LND#2	LND#3	LND#4	LND#5	LND#6	LND#7	LND#8
P01009	Alpha-1-antitrypsin	A1AT	Inhibitor of serine proteases; plays a role in the acute phase response	4.677–5.326	2.896–6.578	0.66	1.11	0.58	0.82	0.69	0.73	1.31	0.59
P04217	Alpha-1B-glycoprotein	A1BG	of unclear specific function, it is thought to be involved in immune system processes and act as a carrier or modulator in cell signaling or inflammation.	0.076–0.295	0.016–0.326	1.27	1.83	**0.09**	0.65	**0.29**	1.08	1.13	1.44
P08697	Alpha-2-antiplasmin	A2AP	Serine protease inhibitor, major targets are plasmin and trypsin. It also inactivates matriptase-3/TMPRSS7 and chymotrypsin.	0.048–0.668	0.000–0.549	**2.14**	1.30	**0.19**	0.73	0.75	**0.46**	**2.27**	**nd**
P02750	Leucine-rich alpha-2-glycoprotein	A2GL	positive regulation of angiogenesis; positive regulation of TGF-β receptor signalling pathway	0.031–0.128	0.000–0.100	0.60	0.77	**nd**	0.70	1.10	**0.42**	0.91	**0.29**
P02765	Alpha-2-HS-glycoprotein	FETUA	Liver-derived plasma protein that regulates calcium homeostasis and inhibits ectopic calcification by binding calcium and phosphate ions.	0.477–0.558	0.212–0.567	0.68	0.92	**0.34**	0.60	0.66	**0.47**	0.79	**0.42**
P01023	Alpha-2-macroglobulin	A2MG	large plasma protein that acts as a broad-spectrum protease inhibitor, trapping and inactivating various enzymes to regulate inflammation and immune responses.	0.000–0.039	0.000–0.056	**3.32**	**2.31**	**0.25**	**2.67**	**nd**	**2.70**	**2.50**	**nd**
P01011	Alpha-1-antichymotrypsin	AACT	serine protease inhibitor that primarily inhibits the enzyme chymotrypsin and other proteases involved in inflammation, thereby protecting tissues from enzymatic damage.	0.308–1.185	0.157–1.245	0.62	0.91	**0.22**	1.71	1.76	0.63	0.66	0.57
P02768	Albumin	ALBU	the most abundant plasma protein, responsible for maintaining oncotic pressure and transporting a wide variety of substances, including hormones, fatty acids, and drugs, throughout the bloodstream.	10.880–16.000	8.655–27.410	0.96	0.88	**2.02**	1.25	0.64	0.87	0.88	0.93
P02760	AMBP protein	AMBP	liver-produced protein that serves as a precursor to two components: alpha-1-microglobulin, which has antioxidant and heme-binding properties, and bikunin, a protease inhibitor involved in inflammation and tissue protection.	0.024–0.047	0.007–0.060	1.00	0.71	0.70	1.52	1.62	1.13	**0.30**	**0.20**
P01019	Angiotensinogen	ANGT	Essential component of the renin-angiotensin system (RAS), a potent regulator of blood pressure, body fluid and electrolyte homeostasis	0.030–0.051	0.028–0.101	0.88	1.34	1.59	0.83	**2.37**	1.04	1.37	0.65
P01008	Antithrombin-III	ANT3	Most important serine protease inhibitor in plasma that regulates the blood coagulation cascade	0.185–0.333	0.110–0.331	1.11	0.88	0.66	1.28	0.79	1.08	1.05	**0.43**
P02647	Apolipoprotein A-I	APA1	Cholesterol homeostasis. May negatively regulate inflammatory response	0.930–1.252	0.600–1.457	1.02	1.38	1.05	1.15	1.37	1.02	1.24	0.57
P06727	Apolipoprotein A-IV	APA4	cholesterol homeostasis; lipid transporter activity; innate immune response in mucosa	0.255–0.690	0.044–0.475	0.62	0.92	**0.37**	1.25	**0.12**	**0.49**	0.58	**0.25**
P02655	Apolipoprotein C-II	APC2	Lipidi binding and cholesterol homeostasis	0.000–0.039	0.000–0.054	**5.51**	0.81	**nd**	**0.22**	**nd**	0.94	**2.65**	**nd**
P02649	Apolipoprotein E	APE	Mediates binding, internalization, and catabolism of lipoprotein particles. May have antioxidant and metal chelating activity	0.000–0.055	0.000–0.075	1.45	1.51	**0.48**	**0.26**	1.14	1.74	**3.55**	**nd**
P09871	Complement C1s subcomponent	C1S	serine protease that plays a key role in the classical complement pathway by cleaving complement proteins C4 and C2, initiating immune responses against pathogens.	0.003–0.026	0.000–0.071	**2.16**	**4.70**	**nd**	**2.94**	**nd**	**2.58**	**3.95**	**nd**
P00450	Ceruloplasmin	CERU	Blue, copper-binding glycoprotein with ferroxidase activity without releasing ROS. Provides iron transport across the cell membrane. Can form complexes with CLUS upon stress	0.081–0.371	0.064–0.403	1.21	1.50	0.54	1.84	0.82	1.18	1.43	**0.29**
P00751	Complement factor B	CFAB	key component of the alternative complement pathway, where it serves to amplify the immune response against pathogens.	0.035–0.238	0.033–0.517	**2.08**	1.36	**3.84**	**2.07**	0.80	1.53	1.21	**0.25**
P05156	Complement factor I	CFAI	Trypsin-like serine protease that plays an essential role in regulating the immune response by controlling all complement pathways.	0.004–0.013	0.000–0.013	1.61	0.89	**nd**	0.51	1.04	1.36	1.32	**nd**
P10909	Clusterin	CLUS	multifunctional glycoprotein involved in lipid transport, cell apoptosis regulation, and clearance of cellular debris, and it also acts as a molecular chaperone protecting cells under stress conditions.	0.023–0.284	0.003–0.228	1.61	0.72	**0.02**	0.52	**0.43**	0.95	1.36	**0.04**
P01024	Complement C3	CO3	Central role in the activation of the complement system promoting inflammation, opsonization, and the formation of the membrane attack complex for immune defense.	0.350–1.252	0.101–0.950	1.05	1.30	0.67	0.82	**0.14**	1.20	0.74	0.71
P0C0L4	Complement C4-A	CO4A	component of the classical and lectin complement pathways that, once activated, contribute to opsonization, inflammation, and the formation of C3 convertase to enhance immune responses.	0.004–0.056	0.005–0.070	0.94	**2.38**	**0.17**	0.73	0.55	**2.43**	1.21	**0.31**
P02741	C-reactive protein	CRP	acute-phase protein that activates the complement system and promotes phagocytosis to aid in immune defense and inflammation.	0.000–0.064	0.000–0.086	**nd**	**nd**	**nd**	**nd**	**4.22**	**3.50**	**2.06**	**nd**
P02671	Fibrinogen alpha chain	FIBA	component of the fibrinogen protein complex that, upon activation by thrombin, helps form fibrin clots to stop bleeding and support wound healing	0.274–1.652	0.040–0.845	1.04	0.99	**0.47**	1.10	**0.05**	1.10	0.88	**0.36**
P02675	Fibrinogen beta chain	FIBB	component of the fibrinogen protein complex that, upon activation by thrombin, helps form fibrin clots to stop bleeding and support wound healing	2.067–3.623	1.195–4.265	0.83	1.24	1.10	**0.42**	1.01	1.51	0.79	1.15
P02679	Fibrinogen gamma chain	FIBG	component of the fibrinogen protein complex that, upon activation by thrombin, helps form fibrin clots to stop bleeding and support wound healing	0.758–1.691	0.670–1.551	0.88	0.64	1.43	1.04	0.86	1.09	1.10	0.62
P68871	Hemoglobin subunit beta	HBB	Involved in oxygen transport from the lung to the various peripheral tissues	0.000–0.252	0.000–0.186	**nd**	**0.18**	**2.19**	0.79	**0.20**	**0.07**	0.52	**0.27**
P02790	Hemopexin	HEMO	glycoprotein that binds free heme with high affinity, preventing oxidative damage and facilitating its transport to the liver for safe degradation and iron recycling	0.501–1.538	0.000–1.768	1.78	**nd**	1.16	0.73	0.73	1.12	1.09	0.93
P00738	Haptoglobin	HPT	binds free hemoglobin released during red blood cell breakdown, preventing oxidative damage and facilitating its clearance by the reticuloendothelial system.	1.361–5.736	0.908–3.462	**0.35**	**0.42**	**0.23**	0.85	**0.22**	0.63	0.83	0.80
P02763	Alpha-1-acid glycoprotein 1 (NA3 glycoform)	A1AG1	NA3 (AGP with 3 sialylated glycan chains) is involved in modulating immune responses, drug binding, and inflammation, and its glycoform profile can change in disease states like cancer, infection, and chronic inflammation.	0.111–0.224	0.108–0.722	0.73	0.77	**2.88**	0.77	**4.89**	1.35	1.67	0.94
P00747	Plasminogen	PLMN	inactive precursor of plasmin, an enzyme that degrades fibrin clots, thereby playing a central role in fibrinolysis and maintaining blood flow after vessel injury.	0.000–0.534	0.000–0.337	1.46	1.48	1.01	0.54	**nd**	1.45	0.99	**nd**
P27169	Serum paraoxonase/arylesterase 1	PON1	enzyme associated with high-density lipoproteins (HDL) that hydrolyze organophosphates and lipid peroxides, contributing to antioxidant defense and cardiovascular protection.	0.000–0.033	0.000–0.026	**nd**	1.25	**nd**	**nd**	**nd**	1.58	**nd**	**nd**
P02753	Retinol-binding protein 4	RETB	Delivers retinol from the liver stores to the peripheral tissues	0.016–0.043	0.009–0.066	0.65	0.96	**0.45**	**2.58**	1.28	1.38	1.58	**0.34**
P02743	Serum amyloid P-component	SAMP	Binds to amyloid fibrils and cellular debris, stabilizing amyloid deposits and participating in the innate immune response through opsonization and complement activation.	0.039–0.097	0.033–0.160	0.51	0.61	0.51	0.82	**2.50**	0.91	1.36	0.81
P00734	Prothrombin	THRB	Functions in blood homeostasis, inflammation and wound healing. Triggers the production of pro-inflammatory cytokines in endothelial cells	0.000–0.120	0.000–0.127	**2.16**	1.70	**0.17**	**nd**	**nd**	1.05	1.38	**nd**
P02787	Serotransferrin	TRFE	glycoprotein that binds and transports iron throughout the body, delivering it to cells via transferrin receptors while maintaining iron homeostasis and limiting free iron toxicity.	1.065–4.117	2.519–3.276	1.05	1.24	1.09	0.96	1.06	1.18	0.97	0.95
P02766	Transthyretin	TTHY	Thyroid hormone-binding protein	0.203–0.733	0.125–0.721	**0.29**	0.56	1.66	0.93	1.11	**0.49**	1.14	1.32
P02774	Vitamin D-binding protein	VTDB	glycoprotein that binds and transports vitamin D and its metabolites in the bloodstream and playing a role in the regulation of calcium homeostasis and immune response.	0.188–0.367	0.118–0.393	0.94	1.12	0.55	1.09	1.13	0.96	1.47	**0.44**
P25311	Zinc-alpha-2-glycoprotein	ZA2G	regulates lipid metabolism, immune response, and inflammation, and it has been implicated in adipocyte differentiation and cancer metastasis.	0.078–0.179	0.000–0.164	1.35	0.55	**nd**	0.69	**nd**	0.78	1.07	**nd**

Protein entry and accession number (AN)a were retrieved from http://www.uniprot.org/. The range of spot% relative volume in control and LND proteome maps is indicated together with fold-change values (ratios of spot% relative volumes in LND/control proteome maps). Values ≥ 2.0 and 0.5 ≤ indicate proteins overrepresented and underrepresented in LND vs. control samples, respectively

Values in bold denote statistically significant differences (*p* < 0.05). Nd: Non detectable

**Fig. 5:**
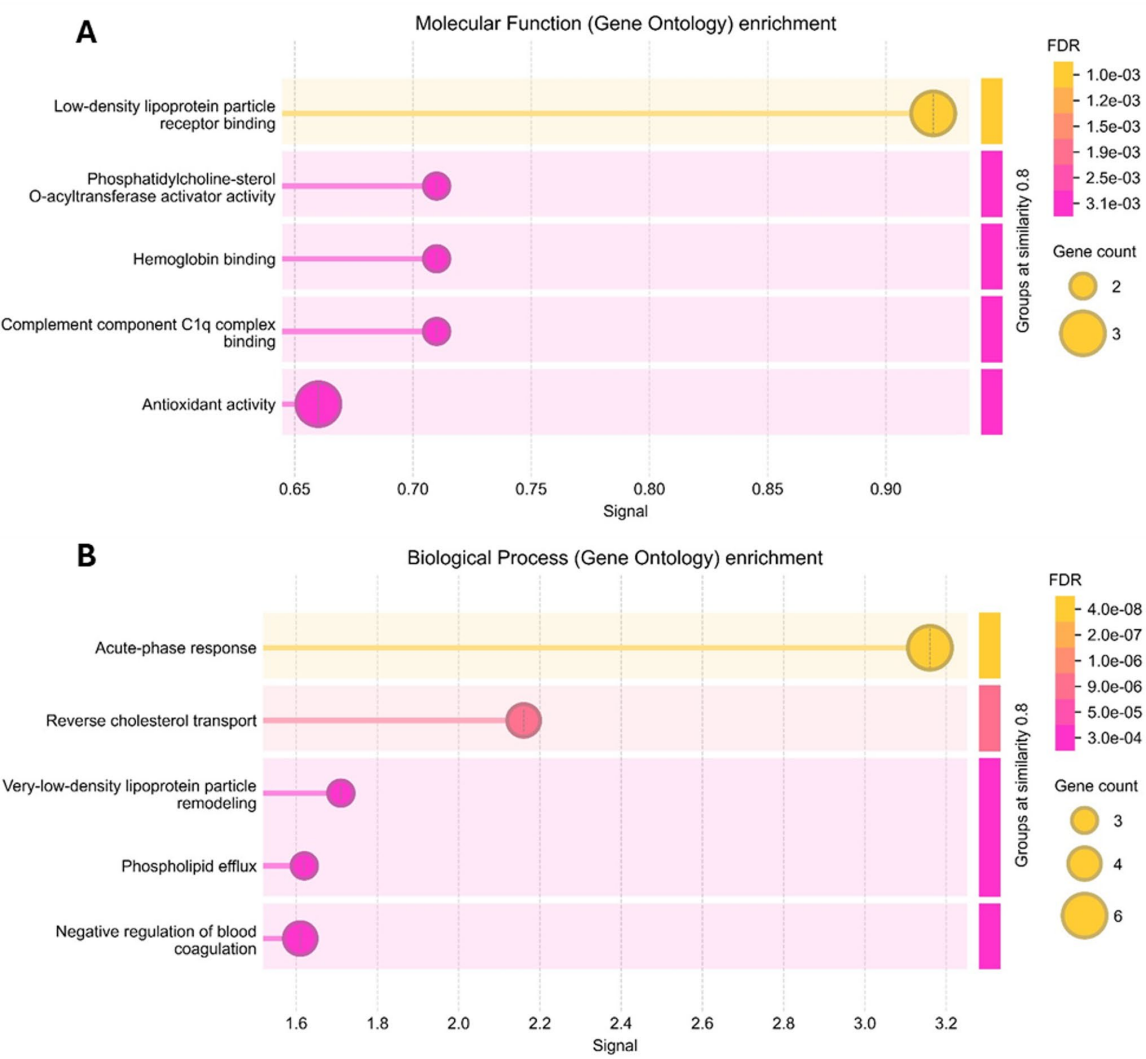
Gene Ontology (GO) enrichment analysis of differentially expressed plasma proteins in LND patients. **A** Top-ranked molecular function categories and **B** biological process categories identified through GO enrichment analysis. Enrichment significance was calculated based on adjusted p-values (FDR < 0.05)

We next applied Gene Ontology (GO) enrichment analysis to the subset of proteins that were differentially expressed in at least three of the eight LND samples. Figure 5 shows the top five GO terms for both molecular function and biological process categories, with “Complement component C1q complex binding” (signal 1.64, FDR 0.00019) and “acute-phase response” (signal 3.58, FDR 9.72E-10) emerging as the highest‐ranking terms, respectively.

Consistent with acute-phase response activation, the abundance of several positive acute-phase proteins was markedly increased in LND plasma, most notably alpha‑2‑macroglobulin, C‑reactive protein, alpha‑2‑antiplasmin, and complement C1s subcomponent (Table 2; Fig. 3). The abundance of classic negative acute-phase proteins such as alpha‑2‑HS‑glycoprotein, apolipoprotein A‑IV, and clusterin was significantly downregulated (Table 2; Fig. 3). Together, these findings corroborate the presence of a sustained, systemic inflammatory status in LND and highlight perturbations in lipid transport and coagulation pathways as additional, potential hallmarks of LND (see Fig. 5).

Haptoglobin (HPT), one of the major acute-phase proteins, was found as under-represented in LND subjects (with a statistically significant lower abundance in four out of the eight LND subjects analyzed here) (Table 2). This protein acts as an antioxidant (binding heme) and is also supposed to play a modulatory and protective role on autoimmune inflammation of the CNS (Galicia et al. 2009) and on the integrity of the nigro-striatal dopaminergic system (Costa-Mallen et al. 2008). Its depletion in LND plasma may therefore reflect, or even contribute to, ongoing neurodegenerative processes. Notably, reduced HPT levels have also been documented in the plasma of Parkinson’s disease (PD) patients, a disorder that shares pathological and clinical overlaps with LND (Zhao et al. 2010).

The increased levels of C1 s subcomponent (C1s) (five out of eight LND subjects), C-FAB (three out of eight LND subjects) and Complement C4-A (two out of eight LND subjects) further supports the activation of the complement system as a contributor to disease progression (Table 2). These findings mirror what observed in proteomic profiling of hyperuricemic patients (Wu and You 2023) and likely arises from inflammasome activation in response to urate crystal deposition (Wu and You 2023).

Finally, we sought to identify overlaps between the proteomic profile of LND and those reported in other dopamine‑related disorders. LND has long been associated with dysfunction of dopamine neurons of the basal ganglia, and strong evidence supports the hypothesis of the developmental origin of such alterations (Witteveen et al. 2022). The dopaminergic system is a complex and powerful neurotransmitter system in the brain. It plays an important regulatory role in motivation, reward, cognition, and motor control. So far, four main dopaminergic pathways have been identified (nigrostriatal, mesolimbic, mesocortical, and tuberoinfundibular) each of which regulates different physiological functions, and imbalances in each pathway are closely related with a variety of diseases such as Parkinson’s disease (PD), schizophrenia (SZ), and autism spectrum disorder (ASD) (Cai et al. 2021; Chen et al. 2024). Over the past decade, proteome techniques were successfully applied to a range of biological samples in the search of biomarkers (with prognostic and diagnostic value) and treatment targets for these DA-related pathologies. To this aim, serum and plasma biospecimens attracted researcher’s attention due to easy accessibility and rapid protein changes in response to disease pathogenesis (Davalieva et al. 2016; Szoko et al. 2017; Dixit et al. 2019). To explore overlap with these disorders, we compared our proteomic data with those reported for three different non-monogenic and non-metabolic diseases affecting different DA pathways, namely PD, ASD and SZ, and partially sharing symptoms with LND. Remarkably, several acute‑phase proteins that were altered in our LND samples, such as alpha‑2‑macroglobulin, apolipoprotein A‑IV, clusterin, alpha‑2‑HS‑glycoprotein, haptoglobin, prothrombin, and retinol‑binding protein 4, transthyretin, serum amyloid P-component, complement factor B and AMBP protein (Table 2) have been reported as dysregulated in PD, SZ, and ASD. This convergence not only reinforces the link between systemic inflammation and dopaminergic dysfunction, but also highlights a shared proteomic signature that may underlie common pathogenic mechanisms across both monogenic and dopamine‑related diseases.

## Conclusions

In this study, we present a preliminary and exploratory proteomic characterization of plasma samples from eight LND individuals, acknowledging that the small cohort size reflects the ultra-rare nature of the disorder (approximately 1:380 000 (Torres 2019). While the limited sample number and methodological constraints preclude definitive conclusions, our combined biochemical and proteomic data provide preliminary evidence of a systemic inflammatory signature in LND, a feature that has not been systematically characterized so far.

To explain these observations, we tentatively propose two non-mutually exclusive mechanisms. First, high UA levels might trigger activation of the NLRP3 inflammasome analogously to what observed in gouty arthritis, where the uptake of monosodium urate crystals deposited in joints by macrophages leads to caspase-1–mediated maturation and release of IL-1β, IL-18, and downstream cytokines (IL-6, TNF-α, IL-33), sustaining and propagating systemic inflammation (Liu et al. 2015). It is tempting to speculate that a similar mechanism may exist in LND. In line with this possibility, despite standard UA- lowering therapy, our LND cohort exhibited higher plasma UA than controls (Table 1), which could contribute to persistent inflammasome activation and the elevated cytokine profile observed. Second, the xanthine oxidase (XO) form of xanthine oxidoreductase may exacerbate oxidative stress: XO-catalyzed oxidation of HYP and XAN to UA generates reactive oxygen species (ROS) as by-products, further promoting inflammatory signaling in LND (Ives et al. 2015; Monteiro et al. 2025). Similar mechanisms have been documented in inflammatory bowel disease (Petrillo et al. 2024) and respiratory virus infections (Pratomo et al. 2021), where an hyperactivation of the NLRP3 inflammasome was correlated with increased XO expression and activity, and support the inhibition of XO as a potential therapeutic target (Pratomo et al. 2021; Petrillo et al. 2024). Although both allopurinol and febuxostat are effective UA-lowering agents, their mechanisms of XO inhibition differ substantially, leading to distinct downstream biological effects. Allopurinol, a purine analog, acts as a competitive substrate inhibitor that binds to the active site of XO and is converted into oxypurinol; in contrast, febuxostat is a non-purine selective inhibitor that stabilizes the enzyme in an inactive conformation resulting in greater suppression of ROS production compared to allopurinol. Accordingly, febuxostat has shown superior suppression of NLRP3-dependent IL-1β release compared to allopurinol in vitro (Monteiro et al. 2025). This supports the notion that the mechanism of inhibition has a central role in the anti-inflammatory capacity of XOR inhibition therapy. As the majority of patients were receiving UA-lowering therapy (allopurinol), treatment status may have partially influenced the observed biochemical and cytokine profiles. This factor should be carefully controlled in future investigations to more accurately delineate disease-specific molecular alterations.

Animal models also support a pro-inflammatory, pro-oxidative role for HYP (Biasibetti et al. 2017; Biasibetti-Brendler et al. 2018). If HYP is administered directly into rat striatum, it activates NF-κB, increases cytokines (IL-6 and TNF-α included), and perturbs antioxidant defenses (ROS, superoxide dismutase, catalase and glutathione peroxidase activity) (Biasibetti et al. 2017) and neuroenergetic parameters (Biasibetti-Brendler et al. 2018).

Although inflammation and oxidative imbalance may theoretically contribute to the neurological manifestations of LND, the present findings do not allow any causal interpretation regarding the relationship between molecular changes and neurological severity. Larger, longitudinal studies integrating clinical neurological assessment with molecular profiling will be essential to explore this potential link.

In summary, our findings offer preliminary, hypothesis-generating insights into possible inflammatory and oxidative processes associated with HPRT deficiency and provide an initial proteomic framework for understanding its molecular basis. Given the exploratory design, modest sample size, and reliance on 2D-PAGE rather than LC–MS/MS proteomics, the detected protein changes should be interpreted as associative rather than causative trends, likely representing downstream consequences of the metabolic and cellular effects of HPRT deficiency. These results are therefore hypothesis-generating and highlight potential molecular pathways that warrant further examination. Future studies involving larger, possibly multi-centre cohorts, and functional validation experiments will be critical to confirm these associations and to clarify whether modulation of inflammatory pathways could, in the long term, represent a therapeutic avenue in LND.

## Supplementary Information

Below is the link to the electronic supplementary material.

Supplementary material 1 (DOCX 1256.3 kb)

## Data Availability

The datasets generated and/or analysed during the current study are not publicly available but are available from the corresponding author on reasonable request.

## References

[CR1] Aebersold R, Mann M (2016) Mass-spectrometric exploration of proteome structure and function. Nature 537(7620):347–355. 10.1038/nature19949 27629641 10.1038/nature19949

[CR2] Biasibetti H, Pierozan P, Rodrigues AF et al (2017) Hypoxanthine intrastriatal administration alters neuroinflammatory profile and redox status in striatum of infant and young adult rats. Mol Neurobiol 54:2790–2800. 10.1007/s12035-016-9866-6 27013467 10.1007/s12035-016-9866-6

[CR3] Biasibetti-Brendler H, Schmitz F, Pierozan P et al (2018) Hypoxanthine induces neuroenergetic impairment and cell death in striatum of young adult Wistar rats. Mol Neurobiol 55:4098–4106. 10.1007/s12035-017-0634-z 28593435 10.1007/s12035-017-0634-z

[CR4] Borsini A, Zunszain PA, Thuret S, Pariante CM (2015) The role of inflammatory cytokines as key modulators of neurogenesis. Trends Neurosci 38:145–157. 10.1016/j.tins.2014.12.006 25579391 10.1016/j.tins.2014.12.006

[CR5] Braconi D, Bernardini G, Paffetti A et al (2016) Comparative proteomics in alkaptonuria provides insights into inflammation and oxidative stress. Int J Biochem Cell Biol. 10.1016/j.biocel.2016.08.016 27590860 10.1016/j.biocel.2016.08.016

[CR6] Braconi D, Bernardini G, Spiga O, Santucci A (2021) Leveraging proteomics in orphan disease research: pitfalls and potential. Expert Rev Proteom 18:315–327. 10.1080/14789450.2021.1918549 10.1080/14789450.2021.1918549 33861161

[CR7] Braconi D, Geminiani M, Psarelli EE et al (2022) Effects of nitisinone on oxidative and inflammatory markers in alkaptonuria: results from SONIA1 and SONIA2 studies. Cells 11:3668. 10.3390/CELLS11223668 36429096 10.3390/cells11223668 PMC9688277

[CR8] Braconi D, Nadwa H, Bernardini G, Santucci A (2025) Omics and rare diseases: challenges, applications, and future perspectives. Expert Rev Proteom 22:107–122. 10.1080/14789450.2025.2468300 10.1080/14789450.2025.2468300 39956998

[CR9] Cai Y, Xing L, Yang T et al (2021) The neurodevelopmental role of dopaminergic signaling in neurological disorders. Neurosci Lett 741:135540. 10.1016/J.NEULET.2020.135540 33278505 10.1016/j.neulet.2020.135540

[CR10] Camici M, Garcia-Gil M, Allegrini S et al (2023) Inborn errors of purine salvage and catabolism. Metabolites. 10.3390/metabo13070787 37512494 10.3390/metabo13070787 PMC10383617

[CR11] Ceballos-Picot I, Mockel L, Potier M-C et al (2009) Hypoxanthine-guanine phosphoribosyl transferase regulates early developmental programming of dopamine neurons: implications for Lesch–Nyhan disease pathogenesis. Hum Mol Genet 18:2317–27. 10.1093/hmg/ddp164 19342420 10.1093/hmg/ddp164 PMC2694685

[CR12] Ceballos-Picot I, Le Dantec A, Brassier A et al (2015) New biomarkers for early diagnosis of Lesch–Nyhan disease revealed by metabolic analysis on a large cohort of patients. Orphanet J Rare Dis 10:7. 10.1186/s13023-014-0219-0 25612837 10.1186/s13023-014-0219-0 PMC4320826

[CR13] Chen H, Li J, Huang Z et al (2024) Dopaminergic system and neurons: role in multiple neurological diseases. Neuropharmacology 260:110133. 10.1016/J.NEUROPHARM.2024.110133 39197818 10.1016/j.neuropharm.2024.110133

[CR14] Christie R, Bay C, Kaufman IA et al (1982) Lesch–Nyhan Disease: clinical experience with nineteen patients. Dev Med Child Neurol 24(4):293–306. 10.1111/J.1469-8749.1982.TB13621.X 7095300 10.1111/j.1469-8749.1982.tb13621.x

[CR15] Costa-Mallen P, Checkoway H, Zabeti A et al (2008) The functional polymorphism of the hemoglobin-binding protein haptoglobin influences susceptibility to idiopathic Parkinson’s disease. Am J Med Genet B Neuropsychiatr Genet 147:216–222. 10.1002/AJMG.B.30593 10.1002/ajmg.b.30593 17918239

[CR16] Cristini S, Navone S, Canzi L et al (2010) Human neural stem cells: a model system for the study of Lesch–Nyhan disease neurological aspects. Hum Mol Genet 19:1939–1950. 10.1093/hmg/ddq072 20159777 10.1093/hmg/ddq072

[CR17] Davalieva K, Kostovska IM, Dwork AJ (2016) Proteomics research in schizophrenia. Front Cell Neurosci 10:1–22. 10.3389/fncel.2016.00018 26909022 10.3389/fncel.2016.00018 PMC4754401

[CR18] Dinasarapu AR, Sutcliffe DJ, Seifar F et al (2022) Abnormalities of neural stem cells in Lesch–Nyhan disease. J Neurogenet 36:81–87. 10.1080/01677063.2022.2129632 36226509 10.1080/01677063.2022.2129632 PMC9847586

[CR19] Dixit A, Mehta R, Singh AK (2019) Proteomics in human Parkinson’s disease: present scenario and future directions. Cell Mol Neurobiol 39:901–915. 10.1007/s10571-019-00700-9 31190159 10.1007/s10571-019-00700-9 PMC11457823

[CR20] Fu R, Chen C-J, Jinnah HA (2014) Genotypic and phenotypic spectrum in attenuated variants of Lesch–Nyhan disease. Mol Genet Metab 112:280–5. 10.1016/j.ymgme.2014.05.012 24930028 10.1016/j.ymgme.2014.05.012 PMC4122630

[CR21] Fu R, Sutcliffe D, Zhao H et al (2015) Clinical severity in Lesch–Nyhan disease: the role of residual enzyme and compensatory pathways. Mol Genet Metab 114:55–61. 10.1016/j.ymgme.2014.11.001 25481104 10.1016/j.ymgme.2014.11.001 PMC4277921

[CR22] Galicia G, Maes W, Verbinnen B et al (2009) Haptoglobin deficiency facilitates the development of autoimmune inflammation. Eur J Immunol 39:3404–3412. 10.1002/EJI.200939291;WGROUP:STRING:PUBLICATION 19795414 10.1002/eji.200939291

[CR23] Göttle M, Prudente CN, Fu R et al (2014) Loss of dopamine phenotype among midbrain neurons in Lesch–Nyhan disease. Ann Neurol 76:95–107. 10.1002/ana.24191 24891139 10.1002/ana.24191 PMC4827147

[CR24] Guibinga G-H, Hsu S, Friedmann T (2010) Deficiency of the housekeeping gene hypoxanthine-guanine phosphoribosyltransferase (HPRT) dysregulates neurogenesis. Mol Ther 18:54–62. 10.1038/mt.2009.178 19672249 10.1038/mt.2009.178 PMC2839227

[CR25] Ives A, Nomura J, Martinon F et al (2015) Xanthine oxidoreductase regulates macrophage IL1β secretion upon NLRP3 inflammasome activation. Nat Commun. 10.1038/ncomms7555 25800347 10.1038/ncomms7555 PMC4382995

[CR26] Jacomelli G, Baldini E, Mugnaini C et al (2019) Inhibiting PNP for the therapy of hyperuricemia in Lesch–Nyhan disease: preliminary in vitro studies with analogues of immucillin-G. J Inherit Metab Dis 42:178–185. 10.1002/JIMD.12039 30740729 10.1002/jimd.12039

[CR27] Javed S, Fersini M, Bernardini G (2024) Unleashing the power of induced pluripotent stem cells in in vitro modelling of Lesch–Nyhan disease. Stem Cell Rev Rep. 10.1007/s12015-024-10821-4 39495466 10.1007/s12015-024-10821-4

[CR28] Jinnah HA, Visser JE, Harris JC et al (2006) Delineation of the motor disorder of Lesch–Nyhan disease. Brain 129:1201–1217. 10.1093/BRAIN/AWL056 16549399 10.1093/brain/awl056 PMC3508431

[CR29] Jinnah HA, Ceballos-Picot I, Torres RJ et al (2010) Attenuated variants of Lesch–Nyhan disease. Brain 133:671–689. 10.1093/brain/awq013 20176575 10.1093/brain/awq013 PMC2842514

[CR30] Kleiner G, Marcuzzi A, Zanin V et al (2013) Cytokine levels in the serum of healthy subjects. Mediators Inflamm. 10.1155/2013/434010 23533306 10.1155/2013/434010 PMC3606775

[CR31] Liu S, Zhou Z, Wang C et al (2015) Associations between interleukin and interleukin receptor gene polymorphisms and risk of gout. Sci Rep 5:1–7. 10.1038/srep13887 10.1038/srep13887 PMC4585862 26399911

[CR32] Lorenzetti M, Bernardini G, Luxbacher T et al (2015) Surface properties of nanocrystalline TiO2 coatings in relation to the in vitro plasma protein adsorption. Biomed Mater (Bristol). 10.1088/1748-6041/10/4/045012 10.1088/1748-6041/10/4/045012 26225819

[CR33] Madeo A, Di Rocco M, Brassier A et al (2019) Clinical, biochemical and genetic characteristics of a cohort of 101 French and Italian patients with HPRT deficiency. Mol Genet Metab 127:147–157. 10.1016/j.ymgme.2019.06.001 31182398 10.1016/j.ymgme.2019.06.001

[CR34] Micheli V, Jacomelli G, Santucci A, Bernardini G (2020) Animal and cell models for Lesch–Nyhan syndrome. Drug Discov Today Dis Models. 10.1016/j.ddmod.2019.10.004

[CR35] Monteiro L, de Archambault B, Starchuk AS LF, et al (2025) Inhibition of Xanthine oxidoreductase with febuxostat, but not allopurinol, prevents inflammasome assembly and IL-1β release. Life Sci Alliance 8:1–11. 10.26508/lsa.202403191 10.26508/lsa.202403191 PMC12095928 40399065

[CR36] Petrillo A, Di, Onali S, Era B et al (2024) P011 altered expression and enzymatic activity of Xanthine oxidase in inflammatory bowel disease. J Crohns Colitis 18:i260–i260. 10.1093/ECCO-JCC/JJAD212.0141

[CR37] Pratomo IP, Noor DR, Kusmardi K et al (2021) Xanthine oxidase-induced inflammatory responses in respiratory epithelial cells: a review in immunopathology of COVID-19. Int J Inflam 2021:1653392. 10.1155/2021/1653392 34367545 10.1155/2021/1653392 PMC8346299

[CR38] Song S, Friedmann T (2007) Tissue-specific aberrations of gene expression in HPRT-deficient mice: functional complexity in a monogenic disease? Mol Ther 15:1432–1443. 10.1038/sj.mt.6300199 17505472 10.1038/sj.mt.6300199

[CR39] Stolp HB (2013) Neuropoietic cytokines in normal brain development and neurodevelopmental disorders. Mol Cell Neurosci 53:63–68. 10.1016/j.mcn.2012.08.009 22926235 10.1016/j.mcn.2012.08.009

[CR40] Sutcliffe DJ, Dinasarapu AR, Visser JE et al (2021) Induced pluripotent stem cells from subjects with Lesch–Nyhan disease. Sci Rep 11:8523. 10.1038/S41598-021-87955-9 33875724 10.1038/s41598-021-87955-9 PMC8055678

[CR41] Szklarczyk D, Kirsch R, Koutrouli M et al (2023) The STRING database in 2023: protein-protein association networks and functional enrichment analyses for any sequenced genome of interest. Nucleic Acids Res 51:D638–D646. 10.1093/NAR/GKAC1000 36370105 10.1093/nar/gkac1000 PMC9825434

[CR42] Szoko N, McShane AJ, Natowicz MR (2017) Proteomic explorations of autism spectrum disorder. Autism Res 10:1460–1469. 10.1002/aur.1803 28509388 10.1002/aur.1803

[CR43] Torres RJ (2019) Current understanding of Lesch–Nyhan disease and potential therapeutic targets. Expert Opin Orphan Drugs 7:349–361. 10.1080/21678707.2019.1652597

[CR44] Torres RJ, Puig JG (2007) Hypoxanthine-guanine phosophoribosyltransferase (HPRT) deficiency: Lesch–Nyhan syndrome. Orphanet J Rare Dis 2:48. 10.1186/1750-1172-2-48 18067674 10.1186/1750-1172-2-48 PMC2234399

[CR45] Torres RJ, Puig JG (2015) Hypoxanthine deregulates genes involved in early neuronal development. Implications in Lesch–Nyhan disease pathogenesis. J Inherit Metab Dis 38:1109–1118. 10.1007/s10545-015-9854-4 25940910 10.1007/s10545-015-9854-4

[CR46] Witteveen JS, Loopstok SR, Ballesteros LL et al (2022) HGprt deficiency disrupts dopaminergic circuit development in a genetic mouse model of Lesch–Nyhan disease. Cell Mol Life Sci. 10.1007/s00018-022-04326-x 35660973 10.1007/s00018-022-04326-x PMC9167210

[CR47] Wu X, You C (2023) The biomarkers discovery of hyperuricemia and gout: proteomics and metabolomics. PeerJ 11:e14554. 10.7717/PEERJ.14554 36632144 10.7717/peerj.14554 PMC9828291

[CR48] Zhao X, Xiao WZ, Pu XP, Zhong LJ (2010) Proteome analysis of the sera from Chinese parkinson’s disease patients. Neurosci Lett 479:175–179. 10.1016/J.NEULET.2010.05.063 20553805 10.1016/j.neulet.2010.05.063

