# Supplementary material for: Uncovering Proteomic and Biochemical Alterations in Plasma from Lesch–Nyhan Disease Patients
Source: Cell Mol Neurobiol. 2025 Dec 15;46:16. doi: 10.1007/s10571-025-01644-z (PMC12804513; doi:10.1007/s10571-025-01644-z)
Supplement: Supplementary file 1 — Supplementary material 1 (DOCX 1256.3 kb) [file 10571_2025_1644_MOESM1_ESM.docx]

**Supplementary**

**Figure 1S:** Distribution and median levels (pg/mL) of cytokines IL-1β, TNF-α, IL-6, IL-18, and IL-33 in LND and LNV subjects grouped according to treatment with allopurinol. Statistical significance was determined with Two-tailed Mann-Whitney test.

**
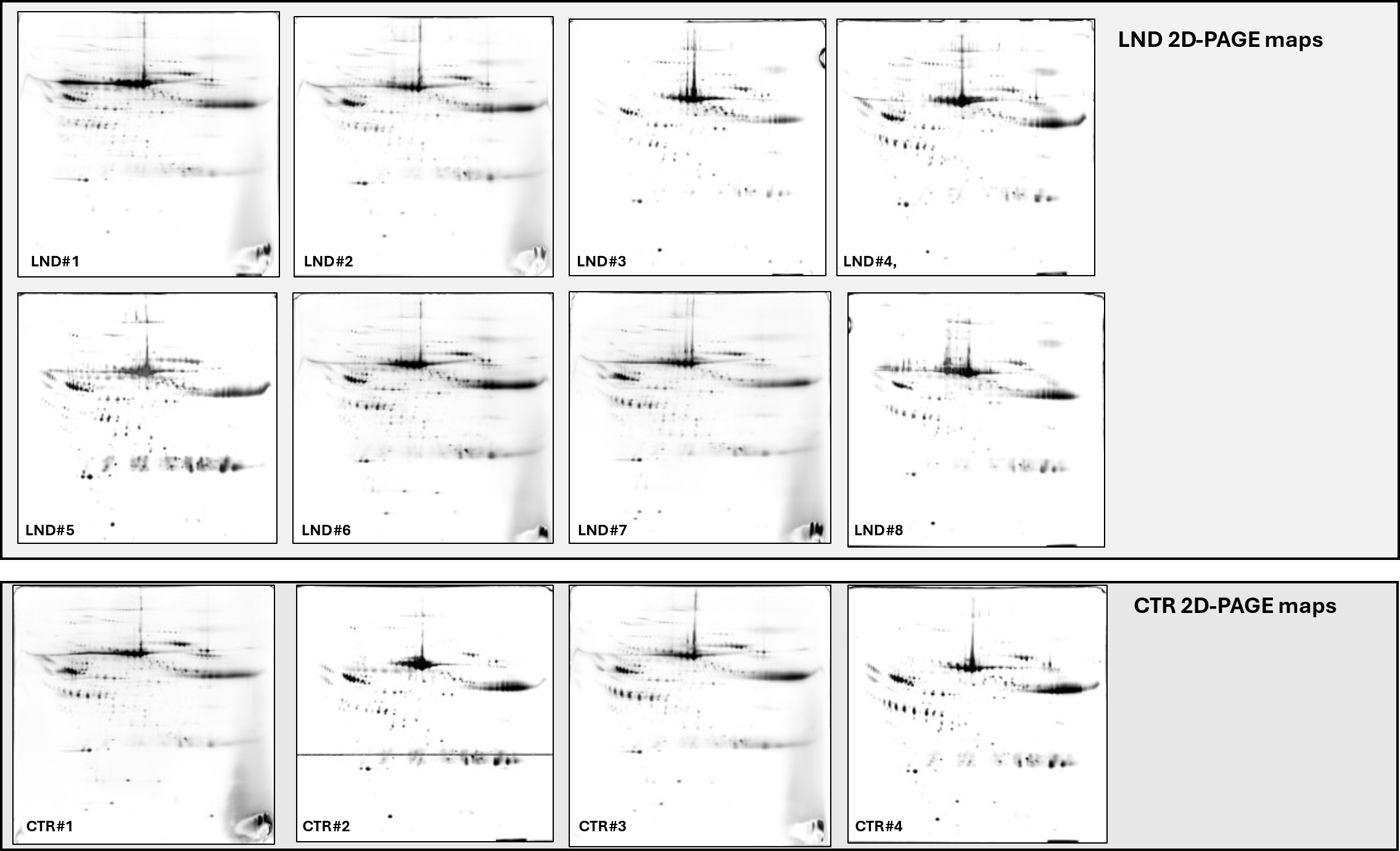
**

**Figure 2S** Comparative proteomics. Silver stained 2D maps of 8 LND plasma samples (from LND#1 to LND#8) and healthy control plasma (from CTR#1 to CTR#4). The quali-quantitative analysis carried out allowed the identification of differently expressed proteins (Figure 3 and Table 3 of the manuscript). Only representative images from a triplicate set are shown.

| **Marker** | **Healthy (N = 20)** | **LND** **(N = 21)** | **LNV** **(N = 8)** | **p value*** |
| --- | --- | --- | --- | --- |
| **AU (µM)** | | | | |
| *min-max* | 129.9-411.8 | 158.1-1783.0 | 174.6-849.6 | ***0.0484****^a^*  *0.6425^b^*  *˃0.9999^c^* |
| *mean±stdev* | 277.2 ± 87.6 | 512.5 ± 396.0 | 420.9±265.7 |  |
| *25-75 %* | 200.8-363.7 | 234.5-620.1 | 223-716.8 |  |
| *median* | 275.8 | 437.6 | 322.7 |  |
| **HYP (µM)** | | | | |
| *min-max* | 0.3-10.0 | 15.9-315.4 | 54.0-169.0 | ***˂ 0.0001^a^***  ***˂ 0.0001^b^***  *˃0.9999^c^* |
| *mean±stdev* | 2.95 ±2.48 | 92.15± 74.08 | 102.70±41.26 |  |
| *25-75 %* | 1.08-4.33 | 40.99-127.80 | 71.91-132.10 |  |
| *median* | 2.36 | 61.68 | 91.65 |  |
| **XAN (µM)** | | | | |
| *min-max* | 0.20-1.32 | 8.9-781.1 | 1.7-402.9 | ***˂0.0001^a^***  ***0.0007^b^***  *˃0.9999^c^* |
| *mean±stdev* | 0.65±0.35 | 171.6 ± 195.5 | 111.8±139.5 |  |
| *25-75 %* | 0.38-0.95 | 35.97-190.5 | 6.163-178.7 |  |
| *median* | 0.525 | 104.7 | 65.85 |  |
| **HPRT (nmol/h/mgHb)** | | | | |
| *min-max* | 81.30-141.9 | 0-1 | 0-21.78 | ***˂0.0001^a^***  ***0.0029^b^***  *0.6232^c^* |
| *mean±stdev* | 111.06±21.75 | 0.21 ± 0.48 | 5.53±10.03 |  |
| *25-75 %* | 91.25-133.7 | 0-0 | 0.002050-16.50 |  |
| *median* | 107.1 | 0 | 0.03 |  |
| **GPRT (nmol/h/mgHb)** | | | | |
| *min-max* | 36.90-146.4 | 0.00-0.60 | 0-0.51 | ***˂0.0001^a^***  ***0.0404^b^***  *0.7372^c^* |
| *mean±stdev* | 101.9±45.13 | 0.046 ± 0.166 | 0.13±0.215 |  |
| *25-75 %* | 50.03-142.3 | 0.00-0.00 | 0-0.29 |  |
| *median* | 117.5 | 0 | 0.07 |  |

**Table 1S. Clinical biochemistry of LND, LNV and healthy controls.** Descriptive statistics (min-max, *mean±stdev,* median and interquartile range) and comparisons of plasma metabolites (UA, HYP, XAN) and enzyme activities (HPRT, GPRT) in healthy age-matched controls, LND, and LNV subjects. One-way ANOVA was performed following normality and log-normality tests. The table includes mean ± standard deviation and p-values calculated using the Kruskal–Wallis test with Dunn’s multiple comparisons. *p-values:^a:^* Healthy vs LND, ^b:^ Healthy vs LNV,^c:^LND vs LNV.

| **Cytokine** | **Healthy** | **LND** | **LNV** | **p value** |
| --- | --- | --- | --- | --- |
| **IL-1β** | | | | |
| *min-max* | 0.83-10.19 | 3.02-33.98 | 3.02-18.45 | **<0.0001*^a^***  **0.0053*^b^***  *˃0.9999^c^* |
| *mean±stdev* | 2.913±2.969 | 11.95± 7.824 | 9.953±6.219 |  |
| *25-75 %* | 0.9250-4.373 | 7.475-12.61 | 3.953-16.78 |  |
| *median* | 1.907 | 10.54 | 8.68 |  |
| *n** | 20 | 21 | 8 |  |
| **IL-6** | | | | |
| *min-max* | 0.1620-26.86 | 5.98-166.2 | 6.5-75.11 | ***˂ 0.0001^a^***  ***˂ 0.0001^b^***  *˃0.9999^c^* |
| *mean±stdev* | 3.235±5.299 | 29.11±39.33 | 31.56±25.19 |  |
| *25-75 %* | 0.85-3.75 | 8.645-27.82 | 7.8-51.41 |  |
| *median* | 1.66 | 12.95 | 28.61 |  |
| *n** | 20 | 21 | 8 |  |
| **TNFα** | | | | |
| *min-max* | 1.44-65.73 | 19.19-115 | 19.39-59.30 | ***˂0.0001^a^***  ***0.0020^b^***  *˃0.9999^c^* |
| *mean±stdev* | 7.977±12.49 | 54.90±25.21 | 41.21±14.41 |  |
| *25-75 %* | 3.518-6.255 | 35.59-72.33 | 30.39-55.22 |  |
| *median* | 4.957 | 47.74 | 40.39 |  |
| *n** | 20 | 21 | 8 |  |
| **IL-18** | | | | |
| *min-max* | 86.91-963.7 | 154.4-1194 | 135.2-742.1 | ***0.0032^a^***  *0.2717^b^*  *0.8944^c^* |
| *mean±stdev* | 245.7±216.1 | 515.9±294.2 | 372.8±190.4 |  |
| *25-75 %* | 121-280 | 272.5-679.5 | 236.4-499.9 |  |
| *median* | 189 | 473.8 | 330 |  |
| *n** | 11 | 21 | 8 |  |
| **IL-33** | | | | |
| *min-max* | 23.46-223.2 | 50.02-3095 | 27.74-9372 | *0.8980^a^*  *0.1209 ^b^*  *0.5303^c^* |
| *mean±stdev* | 127.3±51.6 | 585.9±898.3 | 3661±4165 |  |
| *25-75 %* | 95.4-169.6 | 74.60-590 | 157.6-8031 |  |
| *median* | 121.3 | 161.5 | 1927 |  |
| *n** | 17 | 17 | 5 |  |

**Table 2S. Plasma levels of pro-inflammatory cytokines in LND, LNV, and healthy controls.** Descriptive statistics (min-max, median and interquartile range) are reported for each cytokine: IL-1β, TNF-α, IL-6, IL-18, and IL-33. Group comparisons were performed using the Kruskal–Wallis test, followed by Dunn’s post hoc test for multiple comparisons. Significant differences between groups are indicated where applicable. *p-values:^a:^* Healthy vs LND, ^b:^ Healthy vs LNV,^c:^LND vs LNV. * Note: For some samples, marker concentrations were below the LLOQ (Lower Limit of Quantification); therefore, these samples were excluded from the statistical analysis
